# Differences between human and AI scoring: A meta-analysis of english language assessments

**DOI:** 10.1038/s41598-026-48053-w

**Published:** 2026-04-14

**Authors:** Bingwei Li, Xiao Qunhan, Chunhua Mao

**Affiliations:** 1 College of Foreign Studies, Hunan Normal University, Changsha, China; 2 Hunan Vocational College of Commerce, Changsha, China

**Keywords:** AI scoring, Human scoring, Writing and speaking, Meta-analysis, Scoring differences, Automated assessment, Health care, Mathematics and computing, Psychology, Psychology

## Abstract

Despite a burgeoning body of research on using AI scoring systems in English assessments, concerns regarding their reliability persist. To fill this gap, this meta-analysis examined the AI-human scoring differences and the variables moderating these differences by synthesizing the results of 21 empirical studies with a total of 401,698 participants. Results indicate no statistically significant differences between AI and human scoring; the small effect size implies that the average systematic difference between the two was relatively modest. However, extremely high heterogeneity suggests that this overall finding masks considerable variability across study conditions. Moderator analyses reveal that AI-human scoring differences are significantly influenced by factors such as AI system type, number of human raters, agreement index employed, learner proficiency level (CEFR), and publication year. These findings suggest that while AI cannot fully replace human judgment, it can serve as a diagnostic reference tool within broader quality assurance frameworks. When significant discrepancies arise, they warrant investigation of both scoring sources. Based on these findings, this study offers evidence-based recommendations for educators on the effective use of AI scoring systems in language assessments.

## Introduction

The psychometric challenges inherent in human-mediated assessment of productive skills, i.e., speaking and writing, constitute a persistent dilemma in educational measurement. Their evaluations remain particularly vulnerable to construct-irrelevant variance derived from rater idiosyncrasies^1,2^ because evaluators’ subjective interpretations of scoring rubrics often introduce systematic measurement error^3^. Empirical evidence suggests that even trained raters demonstrate significant scoring discrepancies when assessing complex linguistic constructs such as rhetorical organization or pragmatic competence, particularly in high-stakes testing contexts^4^. While traditional psychometric measures have partially mitigated these issues, for example through standardized scoring procedures in large-scale assessments^5^ and systematic analyses of rater behavior^6,7^, their effectiveness diminishes when applied to context-dependent extended response tasks that require nuanced qualitative judgments. With the rapid advancement of Artificial Intelligence (AI) technologies, educators have begun exploring AI-based scoring systems as innovative tools to address this dilemma. These systems offer distinct advantages, including enhanced objectivity, scalability, and diagnostic precision, emerging as promising tools for tracking learner development and evaluating instructional outcomes more efficiently^8^.

The computational revolution in language assessment, propelled by advances in deep neural architectures and multi-modal learning systems^9^, presents unprecedented opportunities to reconceptualize scoring methodologies. Contemporary AI scoring engines, particularly those employing transformer-based language models, now perform at human level in evaluating surface linguistic features across diverse assessment repertoire—from TOEFL iBT integrated writing tasks to IELTS academic speaking responses^10^. These systems employ multi-dimensional feature extraction techniques covering syntactic complexity, lexical sophistication, and discourse coherence to generate diagnostic profiles surpassing traditional analytic scoring rubrics^11^. However, this technological sophistication also exposes fundamental limitations. State-of-the-art models struggle to decode higher-order communicative constructs like meta-discourse strategies^12^ or interaction competence^13^, where human raters outperform machines on criterion-referenced scales^14,15^. Consequently, doubts persist regarding the extent of differences between AI and human scoring. To fill this void, the present study uses meta-analytic techniques to examine the magnitude of AI-human scoring differences and the factors that influence them^16^^[,17^.

## Literature review

### Prior research on human scoring agreement

The psychometric integrity of human-mediated language assessments hinges on the rigorous quantification of rater agreement, a multi-dimensional construct encompassing interrater reliability, intrarater reliability, and fidelity to scoring criteria^18^. Standard techniques for measuring inter-rater agreement include percentage agreement, *Pearson’s r*, *Spearman’s ρ*, *Cohen’s κ*, and *Fleiss’s κ*^19,20^. In high-stakes assessments, achieving a *Fleiss’ κ* ≥ 0.70 has become the benchmark for ensuring fairness across linguistically diverse populations^5,21^.

Despite moderate-to-strong agreement in standardized contexts (*Cohen’s κ* = 0.60–0.80; *ICC* = 0.75–0.85), particularly for discrete features such as grammatical accuracy and lexical diversity^7,22^, human raters remain susceptible to cognitive biases including halo effects, proximity effects, and primacy effects^1,23^, as well as rubric interpretation drift over time^3,24^. The number of raters also matters: increasing raters generally improves assessment precision^18,25^, though experienced practitioners with similar backgrounds may achieve stable ratings with fewer raters^2^.

AI-based scoring systems offer a partial solution by applying consistent algorithms regardless of sample sequence, thereby minimizing order-related biases^23^. However, AI systems carry their own limitations, including biases inherited from training data^26,27^ and difficulty with construct-relevant features requiring human judgment, such as argumentation depth, rhetorical strategies, and communicative intent^14,15^. These complementary strengths and limitations suggest that combining AI and human scoring may help reduce rater variability while preserving the nuanced evaluation that human raters provide^28^^,29^^,30^^,31^.

### Prior research on AI-human scoring differences and influencing factors

Recently, the application of AI in English assessment has gradually become a focal point. AI scoring tools, including systems based on Natural Language Processing (NLP), Machine Learning (ML), Deep Learning (DL), and Large Language Models (LLMs), can automatically evaluate criterion-specific language products and generate numerical scores or structured feedback^8,32^. Studies indicate that AI systems show small differences from human raters when evaluating surface-level language features such as grammatical errors and coherence, especially in standardized or simplified tasks^10,33^. For instance, in standardized tests like TOEFL iBT and IELTS, AI scoring systems strongly correlate with human raters’ grammar and textual coherence assessments. By extracting multi-dimensional language features, such as academic vocabulary coverage and syntactic complexity, from large-scale textual data, AI systems provide more diagnostic scores, surpassing traditional analytic scoring rubrics^11^. However, the accuracy of AI-generated scores remains limited for complex language tasks. For example, Perelman^34^, demonstrated that AI systems can be misled by surface-level text features, while more recent studies have confirmed that AI-human differences increase with task complexity^14,35^. These concerns have raised ongoing questions about the stability and reliability of AI scoring systems. In particular, doubts remain regarding the differences between AI and human scoring, with multiple factors contributing to these variations^36^^,37^^,38^^,39^.

### Year of publication and customization of scoring systems

Early AI scoring systems were designed explicitly for scoring tasks. For instance, Wang and Brown^40,41^ examined automated essay scoring in educational settings, while Wang and von Davier^42^ and Hoang and Kunnan^43^ evaluated purpose-built engines such as E-rater and MY Access. These systems typically showed small differences from human raters when processing standardized language tasks. At the same time, systems such as E-rater and *Pigaiwang* have demonstrated small differences from human scoring, particularly in evaluating surface-level linguistic features^10,33^. In contrast, although the latest LLMs have made notable progress in natural language processing, they are not designed specifically for scoring tasks, which results in larger differences from human raters^14,35^. Moreover, the type of AI scoring tool significantly impacts the magnitude of differences. For instance, ML systems perform better in simpler tasks, such as evaluating grammar and vocabulary variety^30^. DL and LLMs excel in tasks involving discourse coherence and semantic understanding^44^.

### Task type

As for subjective judgments in writing assessments such as depth of argumentation, discourse coherence, and task-specific communicative intent, AI scoring systems exhibit apparent limitations, and human raters perform more effectively in these areas^14,34^. In speaking assessments, AI systems show small differences in fluency and pronunciation scores but display significant discrepancies with human raters in complex language tasks such as context comprehension and interaction strategies. This highlights the limitations of AI systems in assessing higher-order language abilities, whereas human raters are more effective in these areas, particularly in providing accurate feedback and tracking learners’ progress^15,45^.

### Learner proficiency

Studies show that in higher-level tasks (e.g., CEFR B1-C1), AI systems usually show small differences from human raters, as these tasks are more standardized and the language features are clearer, making it easier for AI models to score^42^. However, for lower-level tasks (e.g., A2-B1), AI systems may struggle to fully understand learners’ communicative intent, resulting in increased scoring discrepancies^46^.

### Sample size

Though research suggests that AI scoring systems typically do not introduce significant bias when the sample size is sufficiently large, AI scoring within small-sample-size contexts may exacerbate scoring differences^26^. This finding further emphasizes the impact of sample size on scoring differences. Due to the larger data set and standardized task requirements, AI systems can produce scores close to human raters in large sample sizes. AI may fail to capture subtle differences in language tasks in smaller sample sizes, leading to increased scoring bias.

### Choice of agreement index

Different agreement indices capture different aspects of scoring differences. *Pearson’s r* measures linear association but does not account for systematic mean-level bias; *Cohen’s Kappa* corrects for chance agreement but may be unstable with skewed score distributions; and Quadratic Weighted Kappa (*QWK*) is sensitive to the magnitude of disagreement but assumes ordinal structure^20,47^. Xu et al.^47^, have argued that the Limits of Agreement (*LOA*) method may provide a more comprehensive picture by capturing both systematic bias and individual-level variability. Given these differing properties, the choice of index can influence how AI-human scoring differences are characterized.

Put together, although a substantial body of research has explored AI-human scoring differences, systematic meta-analyses quantifying these differences and synthesizing multi-dimensional influencing factors remain scarce. Most existing studies focus on a single dimension, lacking comprehensive analysis of multiple variables. Therefore, this study aims to fill this gap by systematically investigating the multi-dimensional factors affecting the magnitude of differences between AI and human scoring, offering new insights for future research and practical recommendations for educators. Specifically, this study is guided by the following questions^48^:

RQ1: To what extent do AI-generated scores differ from human-assigned scores in English language assessments?

RQ2: What factors influence the magnitude of differences between AI and human scoring in language assessments?

RQ3: What are the practical implications of AI-human scoring differences for language assessments?

## Methodology

### Literature search

To ensure a comprehensive synthesis of empirical studies comparing AI-generated scores with human ratings in English assessment, a systematic literature search was conducted following the PRISMA (Preferred Reporting Items for Systematic Reviews and Meta-Analyses) guidelines^49^ and the SMART (Synthesis Methods and Reporting Tool) framework^50^. The search covered the period from October 2007 to January 2025, encompassing the evolution of AI scoring technologies from early rule-based engines to advanced transformer-based LLMs^51^^,52^^,53^.

Four major academic databases were selected to maximize coverage of English- and Chinese-language publications: CNKI (China National Knowledge Infrastructure), Google Scholar, ResearchGate, and Web of Science. This bilingual approach reflects the global implementation of AI scoring systems in English education.

Search terms were constructed based on three conceptual domains: scoring mechanism, scoring agent, and assessment type. These were combined using Boolean operators. The full search string applied to English-language databases (Google Scholar, ResearchGate, and Web of Science) was: (AI scoring OR automated scoring OR machine scoring OR automated essay scoring OR automated writing evaluation) AND (human scoring OR manual scoring OR human rater OR expert rater) AND (English writing assessment OR speaking assessment OR language testing OR writing evaluation OR essay scoring). For CNKI, equivalent Chinese terms were used: (人工智能评分 OR 自动评分 OR 机器评分) AND (人工评分 OR 手动评分 OR 人工评阅) AND (“英语写作评估 OR 口语评价 OR 英语语言测试). The search was limited to peer-reviewed journal articles, book chapters, and conference proceedings published between October 2007 and January 2025. No restrictions were applied to study design or geographic origin. The initial database searches were conducted between November 2024 and January 2025, with a final update search performed on May 4, 2025 to capture any newly published studies. All searches were restricted to title, abstract, and keyword fields where the database permitted such filtering^54^^,55^^,56^.

**Fig. 1:**
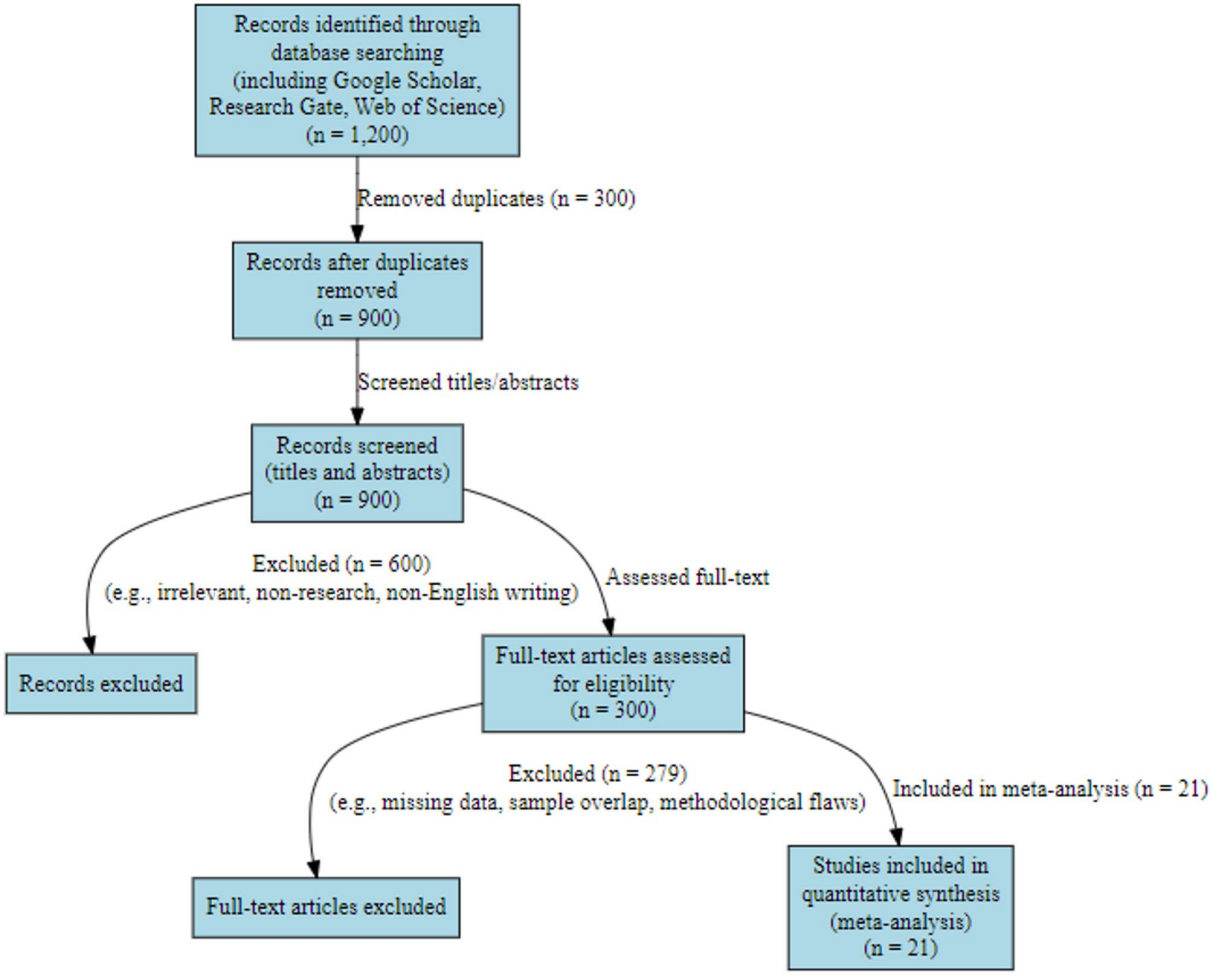
Literature search and selection processes.

Search queries were refined iteratively to improve recall and precision. The final search retrieved 1,200 records—896 in English and 304 in Chinese. After removing 300 duplicates, 900 unique records were retained for screening. Titles and abstracts were first reviewed for relevance, resulting in 300 articles (201 English, 99 Chinese) selected for full-text examination. Of these, 21 studies met all inclusion criteria and were included in the final meta-analysis. The screening and selection process is summarized in Fig. 1.

### Inclusion and exclusion criteria

To ensure the validity, reliability, and comparability of studies included in this meta-analysis, stringent inclusion and exclusion criteria were developed based on the research questions we proposed and then applied during the iterative screening process. These criteria were designed to capture empirical studies on differences between AI and human scoring in English assessments. Studies were included if they met the following criteria^57^^[,58^^[,59^:

1. Topical Relevance: The study must directly examine automated scoring systems and compare their output with human rater scores in English writing and/or speaking assessments.
2. AI Scoring System: The automated scoring tool must utilize NLP, ML, DL, LLMs techniques, and be capable of generating evaluative feedback or numerical scores without human intervention.
3. Outcome Measures: The study must report quantitative outcomes from AI and human raters, with sufficient statistical information to compute or convert effect sizes (e.g., means, standard deviations, sample sizes, correlations).
4. Research Design: Eligible designs include experimental, quasi-experimental, and correlational studies.
5. Methodological Quality: Studies must demonstrate sufficient methodological rigor, including clear research questions, transparent scoring procedures, adequate sample sizes, and appropriate statistical analyses.
6. Publication Characteristics: Studies must be published in peer-reviewed journals, academic books, book chapters, or conference proceedings, with accessible full texts and clearly reported methods.
7. Ethical Standards: Studies must comply with basic ethical principles, including informed consent, protection of participant data, and institutional approval where relevant.

Studies were excluded if they met any of the following conditions:

1. The study was theoretical in nature, including literature reviews, editorials, or methodological commentaries, and lacked empirical scoring data;
2. The focus was solely on algorithmic optimization or model development without a comparative analysis of human and AI scoring outcomes;
3. The study failed to report sufficient statistical information necessary for the calculation of effect sizes;
4. The study lacked transparency in critical areas such as sample characteristics, task descriptions, or rating procedures;
5. The study consisted of duplicates, poster abstracts, or short notes with inadequate methodological detail;
6. The study exhibited low methodological rigor, as indicated by any of the following: (a) a sample size fewer than 10 participants; (b) poor internal consistency; (c) validity concerns in the scoring comparison, operationally defined as the absence of the use of untrained or uncalibrated human raters, or failure to report essential scoring procedures.
7. The study presented ambiguous or contradictory findings without appropriate interpretation or clarification.

Ultimately, 21 studies met all inclusion criteria and were retained for quantitative synthesis^60^^,61^^,62^.

### Coding procedure

All 21 studies included in this meta-analysis were independently coded by two trained researchers based on a standardized coding protocol derived from the PRISMA guidelines^49^. Both coders held doctoral-level training in language assessment and received a two-week intensive training on the coding manual, including multiple sessions on interpreting complex scoring indices and extracting psychometric data. Prior to formal coding, a pilot coding round was conducted using five representative studies to test the coding scheme’s clarity, consistency, and feasibility.

During the pilot phase, the coders collaboratively refined the operational definitions of key variables, ultimately establishing a coding scheme comprising seven distinct categories. As detailed in Table 1, these categories were consistently extracted from each included study^63^^,64^:

1. Study Identification: Author(s) and year of publication.
2. Scoring Outcomes: Mean scores, standard deviations, and sample sizes to calculate the Cohen’s d as the primary effect size.
3. Human Rating Characteristics: Number of human raters involved in the assessment process.
4. Learner Proficiency Level: CEFR (Common European Framework of Reference for Languages) level of participants as reported in each study.
5. Assessment Task: Type of task assessed to explore task-specific effects on scoring differences.
6. AI Type: Type of AI model used to examine the impact of technological advancements on scoring differences.
7. Type of Agreement Index: Primary agreement indices reported in each study.

These categories were inductively derived through preliminary examination of all 21 studies and were cross-referenced with existing frameworks in language assessment meta-analyses (e.g^18,65^).

To evaluate inter-coder reliability, *Cohen’s Kappa* was computed for each of the seven coding dimensions. Acceptable agreement was defined as *κ* ≥ 0.80, based on commonly accepted thresholds^66^. The average *κ* across all categories was 0.88, with individual categories ranging from 0.83 to 0.92, indicating high reliability. Discrepancies were resolved through discussion, and the third expert adjudicated in cases of persistent disagreement. This rigorous process ensured accurate, reliable, and replicable data extraction.

Notably, the category 2 served as the basis for addressing RQ1, whereas categories 1 and 3–7 were used to identify potential moderators for RQ2.

### Calculating effect sizes

This meta-analysis used *Cohen’s d* as the primary effect size indicator to estimate the magnitude of systematic differences between AI and human scoring in English assessments. For studies reporting means and standard deviations from both scoring sources, *Cohen’s d* statistics were computed following the procedures outlined by Morris and DeShon^67^.

In cases where studies reported alternative statistics, these were converted into equivalent *d* values using established meta-analytic conventions to ensure comparability across studies. Positive effect sizes indicate that AI-generated scores were generally higher than human raters, whereas negative values suggest that AI tended to assign lower scores than humans.

It should be noted that *Cohen’s d* quantifies systematic mean differences between AI and human scores but does not directly assess scoring agreement or reliability. Two sets of scores can yield a small d while still exhibiting substantial individual-level disagreement, as *Cohen’s d* is insensitive to within-pair variability. Agreement-based metrics such as *ICC* would more directly capture concordance between scoring sources. However, the primary studies reported heterogeneous agreement indices, making it infeasible to conduct a unified agreement-based meta-analysis. *Cohen’s d* was selected because it could be computed or converted from the most commonly available statistics across all included studies, thereby maximizing comparability.

**Table 1:** Summary of Included Studies.

No.	Authors	Year	Machine			Human			No. of Human Raters	CEFR Level	Task Type	AI Type	Type of Agreement Index
			M	SD	*N*	M	SD	*N*					
1	Sun & Zhang	2020	6.02	1.5	206	5.72	1.5	206	3	B2	Speaking	ML	*Pearson*
2	Vo et al.	2023	85.5	10.2	37,000+	87.2	9.8	37,000+	10–15	A2–B1	Writing	ML	*Pearson*
3	Wilson & Huang	2024	18.09	4.87	2221	16.64	4.74	2221	9	A2–B1	Writing	NLP + ML	*Kappa*
4	Yamashita	2024	75.4	7	136	76.5	6.9	136	80	A2–B2	Writing	LLM	*QWK*
5	Uyar & Büyükahıska	2025	5.5	1.1	50	7.3	1.2	50	3	B1–B2	Writing	LLM	*Spearman*
6	Wang & Brown	2008	5.98	0.87	107	5.22	0.96	107	10–15	B1–B2	Writing	NLP	*Spearman*
7	Wang & Brown	2007	5.98	0.87	107	5.22	0.96	107	2	B1–B2	Writing	NLP	*ICC*
8	Wang & von Davier	2014	3.45	1.12	352,895	3.08	1.22	352,895	10–15	B1–C1	Writing	NLP	*Kappa*,
9	Topuz et al.	2024	2.61	0.69	210	3.64	0.71	210	2	B1–B2	Writing	LLM	*Pearson*
10	Tang et al.	2024	2.61	0.69	210	3.64	0.71	210	2	A2–B1	Writing	LLM	*QWK*
11	Hoang&Kunnan	2016	4.09	1.19	147	3.76	1.18	147	2	B1–B2	Writing	ML	*Pearson*
12	Zribi & Smaoui	2021	4.44	0.77	15	3.38	0.71	15	10	B1–B2	Writing	ML	*ICC*
13	Correnti et al.	2019	75.5	10.2	1529	78.3	9.7	1529	14	A2–B1	Writing	NLP	*ICC*
14	Zhao et al.	2023	6.473	2.045	3750	6.326	2.085	3750	4	A2–B1	Writing	NLP + ML	*QWK*
15	Bui & Barrot	2025	14.07	2.5	200	16.79	2.5	200	1	A2–B2	Writing	LLM	*ICC*
16	Chen & Pan	2022	86.6	4.71	30	84.87	4.44	30	5	B1–B2	Writing	DL + NLP + ML	*Pearson*
17	Wang & Bai	2021	7.92	1.8	486	8.77	1.95	486	2	B1–B2	Writing	ML + NLP	*Pearson*
18	Jin et al.	2020	10.23	1.02	36,288	10.05	1.24	36,288	2	B1–B2	Speaking	DL	*Pearson*
19	Zhang & Zhu	2022	9.36	3.46	2902	9.17	4.39	2902	12	B1–B2	Writing	DL	*Kappa*
20	Bai & Wang	2018	7.92	1.8	150	8.77	1.95	150	10–15	B1–B2	Writing	ML	*Pearson*
21	Lu	2022	60.78	14.27	59	68.58	16.46	59	2	B1–B2	Speaking	NLP	*Pearson*

### Statistical analysis

This meta-analysis used the Metafor package in R Studio to analyze data extracted from 21 studies (see Table 1). Extracted variables included sample sizes, mean scores, standard deviations, publication year, number of human raters, CEFR level, task type, AI type, and type of agreement index, following the SMART framework for transparency^50^.

To address RQ1, *Cohen’s d* was calculated as the primary effect size using the escalc function in Metafor. A random-effects model with Restricted Maximum Likelihood (REML) was employed to aggregate effect sizes across studies^68^, accounting for both within-study and between-study variance. The overall effect size and its 95% confidence interval were calculated to determine the magnitude and statistical significance of scoring differences.

To address RQ2, heterogeneity was first assessed using *tau²* (between-study variance), *I²* (percentage of total variance due to heterogeneity), and *Q* statistics (test of homogeneity). When significant heterogeneity was detected (*p* <.05), meta-regression analyses were conducted using the rma function to examine moderating effects of publication year, AI type, task type, CEFR level, number of raters, and agreement index type. Meta-regression coefficients represent the change in effect size per unit change in the moderator, with 95% confidence intervals indicating the precision of these estimates. Subgroup analyses were additionally performed to compare effect sizes across categorical moderators.

Publication bias was assessed using Egger’s test and funnel plots, with the Trim-and-Fill method applied when bias was detected (*p* <.05). Sensitivity analyses excluded small-sample studies (*N* < 100) to evaluate the robustness of the results.

## Results

### Overall effect size

Table 2 summarizes the main results of the present meta-analysis, including effect sizes, standard errors, confidence intervals, and the number of included studies. The overall effect size (*Cohen’s d*) for the difference between AI and human scoring was − 0.1444 (*SE* = 0.1633, z = −0.8841, *p* =.3767). The results indicate no statistically significant difference between AI and human scoring. According to the interpretive thresholds proposed by Plonsky and Oswald^65^, the observed effect size falls within the “small” range (|*d*| < 0.2), which suggests that the average difference between AI and human scores is relatively modest.

**Table 2:** Random Effects Model Results: Meta-Analysis of AI and Human Scoring in English Writing and Speaking Assessments.

Metric	Estimate/Result	Standard Error (SE)	Statistic	*p*-value	95% Confidence Interval
Overall Effect Size (*Cohen’s d*)	−0.1444	0.1633	*z* = −0.8841	0.3767	[−0.4644, 0.1757]
Heterogeneity (*tau*^*2*^)	0.5403	0.1769	-	< 0.0001	-
*I*^*2*^ (% Heterogeneity)	99.95%	-	-	< 0.0001	-
*H*^*2*^ (Variance Ratio)	2008.99	-	-	< 0.0001	-
*Q* Statistic (*df* = 20)	5431.7692	-	-	< 0.0001	-
Model Fit	logLik = −22.9718, deviance = 45.9435, AIC = 49.9435, BIC = 51.9350, AICc = 50.6494	-	-	-	-

### Heterogeneity analysis

A heterogeneity analysis was performed to determine whether the overall effect size varied significantly across studies. This step was essential for identifying differences in study characteristics that could impact the magnitude of scoring differences^69^. As shown in Table 2, the *I²* value of 99.95% indicates that the observed variation is primarily due to heterogeneity between studies rather than sampling error. The *tau²* value of 0.5403 suggests a high degree of variability in scoring differences across studies, and the standard error (*SE*) of 0.1769 reflects the uncertainty in the estimation. The *Q* statistic of 5431.7692 (*p* <.0001) further confirms the significance of the heterogeneity.

This high level of heterogeneity suggests that AI-human scoring differences vary considerably across study conditions. The moderator analyses and subgroup comparisons in the following sections are therefore more informative than the overall pooled estimate for understanding when and to what extent AI and human scores diverge.

### Meta-regression analysis

The results of meta-regression analysis are presented in Table 3. Publication year showed a significant negative association with effect size (*β* = −0.0831, *p* =.0313), indicating that scoring differences increased over time, with the effect size shifting by − 0.0831 for each additional year.

In analyzing AI system types, models combining NLP + ML (*β* = 1.0211, *p* =.0341) or DL + NLP + ML (*β* = 1.4238, *p* =.0374) showed significantly larger positive effect sizes relative to the reference category, indicating that these multi-component systems tended to produce AI scores higher than human scores. In contrast, ML and LLM did not significantly differ from the reference category.

Number of raters emerged as a significant moderator (*β* = 0.0157, *p* =.0115), indicating that as the number of raters increases, the difference between AI scores and human scores decreases.

Regarding the agreement index used, studies employing *Pearson’s r* reported significantly lower effect sizes (*β* = −0.7428, *p* =.0267), while *Cohen’s Kappa*, *QWK*, *ICC*, and *Spearman’s ρ* did not significantly moderate the effect size. No significant moderating effects were found for task type or CEFR level.

**Table 3:** Meta-Regression Analysis by AI Type, Task Type, CEFR Level, and Agreement Index.

Variable	Estimate	SE	z-value	*p*-value	Confidence Interval (95%)
Intercept	0.2380	0.2401	0.9915	0.3214	[−0.2325, 0.7086]
Year	−0.0831	0.0386	−2.1531	0.0313	[−0.1588, −0.0075]
AI_Type_ML	0.7564	0.3892	1.9432	0.0520	[−0.0065, 1.5193]
AI_Type_LLM	−0.4018	0.5226	−0.7688	0.4420	[−1.4261, 0.6225]
AI_Type_DL	0.5399	0.3772	1.4313	0.1523	[−0.1994, 1.2792]
AI_Type_NLP + ML	1.0211	0.482	2.1185	0.0341	[0.0764, 1.9658]
AI_Type_DL + NLP + ML	1.4238	0.6839	2.0819	0.0374	[0.0834, 2.7643]
AI_Type_ML + NLP	0.562	0.6133	0.9164	0.3594	[−0.6400, 1.7641]
Task_Type_Writing	−0.4813	0.4365	−1.1027	0.2701	[−1.3368, 0.3742]
CEFR_Numeric	0.095	0.3279	0.2897	0.7720	[−0.5478, 0.7378]
N_Raters	0.0157	0.0062	2.5278	0.0115	[0.0035, 0.0278]
Agreement_Index_Pearson	−0.7428	0.3351	−2.2164	0.0267	[−1.3997, −0.0859]
Agreement_Index_Kappa	−0.2244	0.4186	−0.5361	0.5919	[−1.0448, 0.5960]
Agreement_Index_QWK	−0.4367	0.3886	−1.1239	0.2611	[−1.1982, 0.3249]
Agreement_Index_Spearman	−0.3121	0.37	−0.8435	0.3989	[−1.0374, 0.4131]

### Publication bias

Publication bias was assessed using Egger’s regression test, which statistically evaluates funnel plot asymmetry by regressing the standard normal deviate on study precision. A significant intercept indicates potential bias due to over-representation of studies with statistically significant results^70^.

The results of Egger’s test (see Table 4) showed that publication bias did not reach statistical significance (*z* = −1.2315, *p* =.2171). However, due to the limited number of studies, the statistical power may not be sufficient to detect minor biases.

**Table 4:** Publication bias analysis of AI and Human Scoring in English writing and speaking assessments.

Metric	Estimate/Result	Statistic	*p*-value
Egger’s Test (Intercept)	−1.2345	*z* = −1.2345	0.2171

**Fig. 2:**
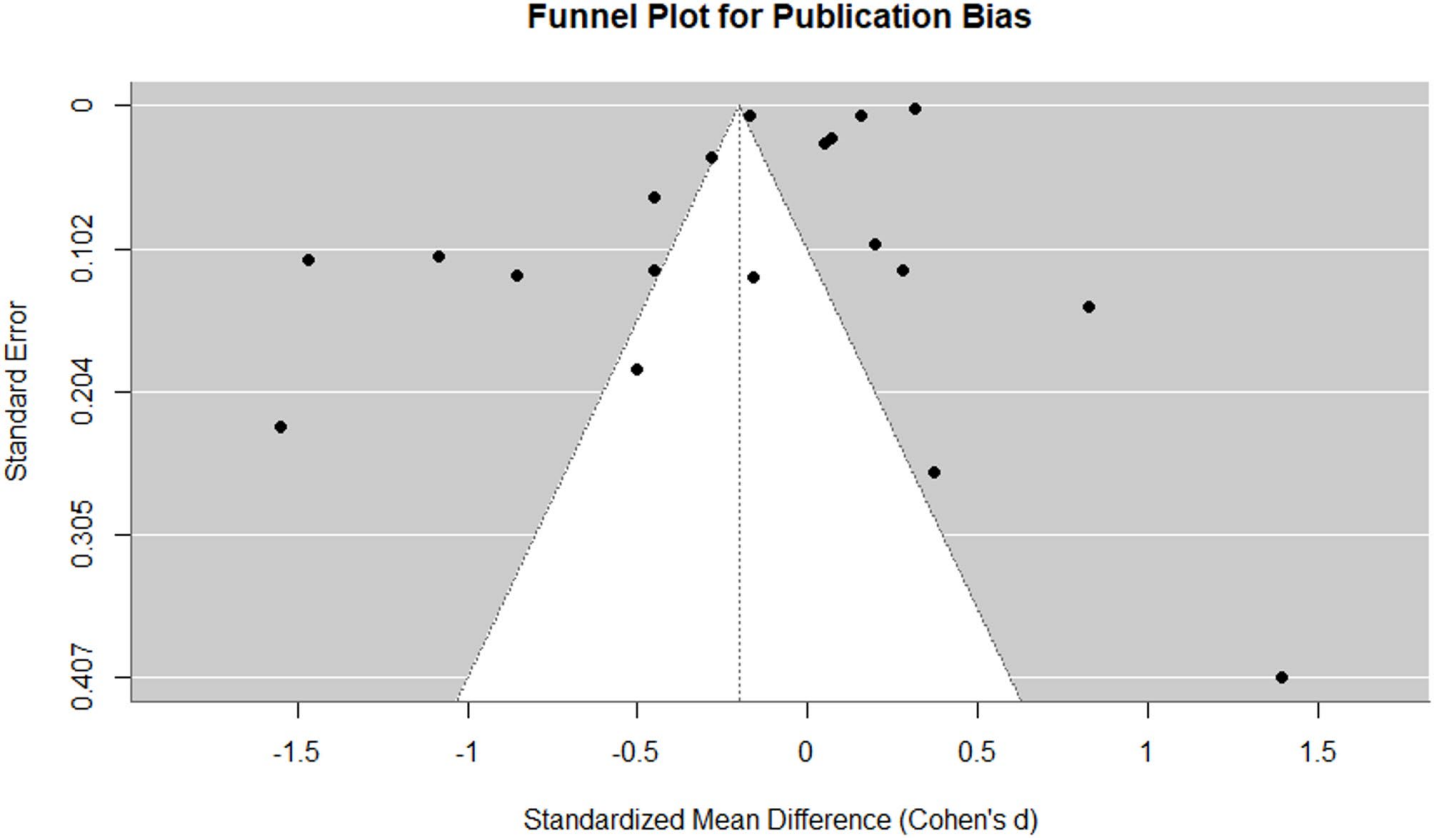
Original funnel plot of AI and human scoring in English writing and speaking assessments.

Figure 2 shows an asymmetric distribution of effect sizes. Small-sample studies (*SE* = 0.3–0.4) are concentrated in the negative range (*d* = − 1.0 to − 0.5), while large-sample studies (*SE* = 0.1–0.2) cluster near zero. However, the presence of large-sample outliers such as *N* = 352,895^42^ indicates that the dispersion cannot be fully attributed to small-sample effects. This pattern warrants cautious interpretation, as explored further in the sensitivity analysis below.

### Sensitivity analysis

To assess the robustness of the overall effect size, a sensitivity analysis was performed by excluding five studies with small sample sizes (*N* < 100). The adjusted effect size was − 0.0876 (*SE* = 0.1421, *p* =.0378), smaller than the full-sample estimate, suggesting that small-sample studies meaningfully influenced the overall result. However, heterogeneity remained high (*I²* = 99.92%, *Q* = 4987.2345, *p* <.0001), indicating that sample size differences account for only part of the variability.

**Fig. 3:**
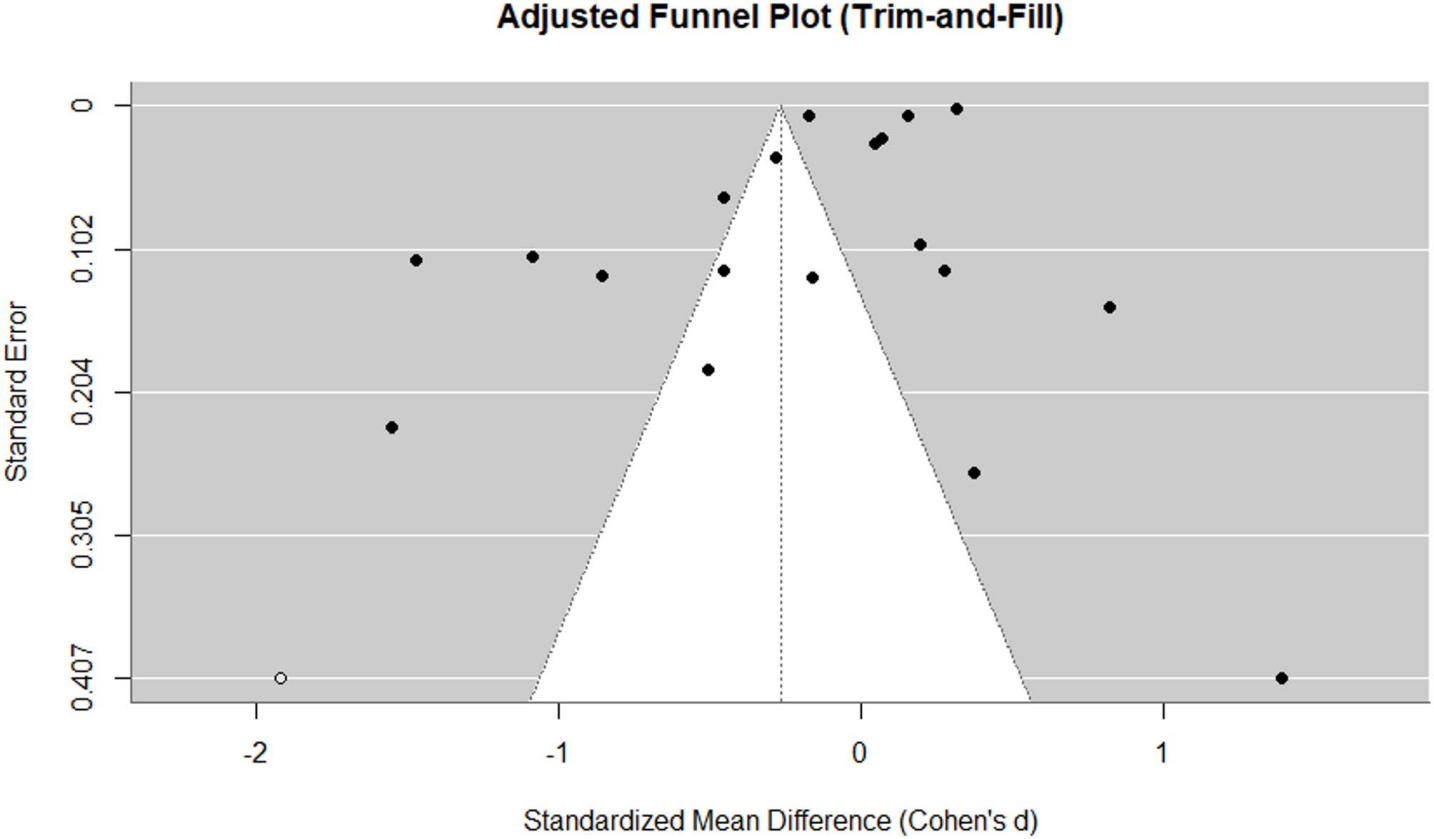
Adjusted funnel plot of AI and human scoring in English writing and speaking assessments.

The trim-and-fill method (Fig. 3) identified eight potentially missing studies in the positive effect size region. After adjustment, the overall effect size shifted to 0.0312 (*p* =.7896), remaining non-significant. The wide confidence interval and persistent heterogeneity suggest that scoring differences are driven primarily by study-level moderators rather than publication bias.

### Subgroup analysis

**Fig. 4:**
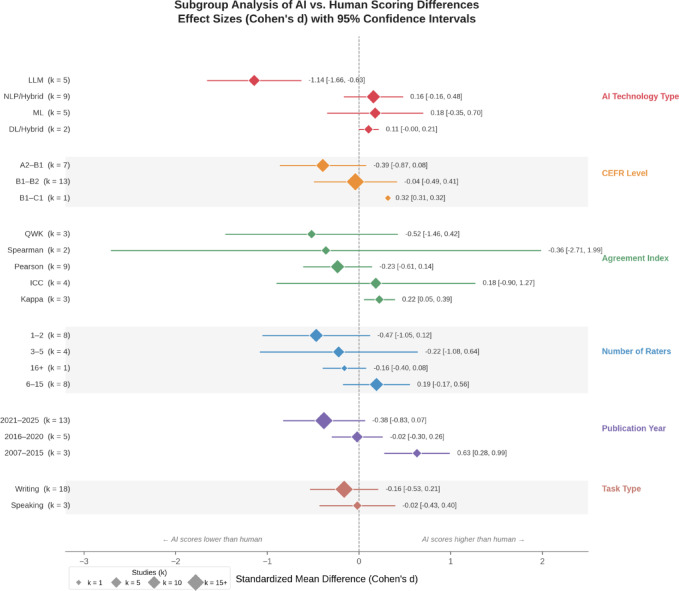
Subgroup Analysis Forest Plot of AI vs. Human Scoring Difference.

The results of subgroup analysis are presented in Table 5 and Fig. 4. The studies were categorized into three periods: 2007–2015, 2016–2020, and 2021–2025. During 2007–2015, the effect size was 0.6337 (*p* =.0005), indicating that AI-generated scores were significantly higher than human scores. Between 2016 and 2020, the effect size was − 0.0195 (*p* =.0013); although statistically significant, the near-zero value suggests negligible practical differences. In the 2021–2025 period, the effect size was − 0.3811 (*p* =.0946), larger in magnitude than the preceding period but not statistically significant, suggesting that AI-human scoring differences may have increased in recent years, likely reflecting the inclusion of general-purpose LLMs in this time period.

**Table 5:** Subgroup Analysis of AI and Human Scoring Differences.

Variable	Subgroup	*N* Studies	Effect Size	Confidence Interval	*P* Value	Direction
AI_Type	DL/Hybrid	2	0.1062	[−0.0019, 0.2143]	0.0542	Machine > Human
AI_Type	LLM	5	−1.1431	[−1.6572, −0.6289]	0	Human > Machine
AI_Type	ML	5	0.1764	[−0.3485, 0.7012]	0.5102	Machine > Human
AI_Type	NLP/Hybrid	9	0.1586	[−0.1649, 0.4821]	0.3365	Machine > Human
CEFR_Level	A2-B1	7	−0.3937	[−0.8651, 0.0777]	0.1017	Human > Machine
CEFR_Level	B1-B2	13	−0.0399	[−0.4934, 0.4136]	0.863	Human > Machine
CEFR_Level	B1-C1	1	0.3160	[0.3113, 0.3206]	0	Machine > Human
Agreement_Index	*ICC*	4	0.1846	[−0.9002, 1.2694]	0.7388	Machine > Human
Agreement_Index	*Kappa*	3	0.2226	[0.0525, 0.3928]	0.0103	Machine > Human
Agreement_Index	*Pearson*	9	−0.2329	[−0.6093, 0.1435]	0.2253	Human > Machine
Agreement_Index	*QWK*	3	−0.5166	[−1.4579, 0.4247]	0.2821	Human > Machine
Agreement_Index	*Spearman*	2	−0.3604	[−2.7058, 1.985]	0.7633	Human > Machine
Number_of_Raters	1–2	8	−0.4660	[−1.0532, 0.1212]	0.1198	Human > Machine
Number_of_Raters	16+	1	−0.1583	[−0.3963, 0.0798]	0.1925	Human > Machine
Number_of_Raters	3–5	4	−0.2205	[−1.0821, 0.6411]	0.6159	Human > Machine
Number_of_Raters	6–15	8	0.1905	[−0.1747, 0.5556]	0.3066	Machine > Human
Overall All_		21	−0.1456	[−0.4671, 0.176]	0.3750	Human > Machine
Publication_Year	2007–2015	3	0.6337	[0.2765, 0.9909]	0.0005	Machine > Human
Publication_Year	2016–2020	5	−0.0195	[−0.2986, 0.2597]	0.0013	Human > Machine
Publication_Year	2021–2025	13	−0.3811	[−0.8281, 0.0658]	0.0946	Human > Machine
Task_Type	Speaking	3	−0.0180	[−0.4314, 0.3953]	0.9319	Human > Machine
Task_Type	Writing	18	−0.1613	[−0.5343, 0.2117]	0.3967	Human > Machine

The analysis categorized AI system types into four subgroups: DL/Hybrid, LLM, ML, and NLP/Hybrid. LLM showed the largest effect size (*d* = − 1.1431, *p* <.0001, *k* = 5), indicating that human scores were significantly higher than AI scores. However, the small number of studies in this subgroup limits the generalizability of this finding. DL/Hybrid systems showed a marginally non-significant AI advantage (*d* = 0.1062, *p* =.0542). No significant differences were observed for ML (*d* = 0.1764, *p* =.5102) or NLP/Hybrid (*d* = 0.1586, *p* =.3365), indicating no systematic differences between these AI systems and human ratings.

The CEFR level analysis revealed some variation across proficiency levels. For B1–C1 levels, AI scores were significantly higher than human scores (*d* = 0.3160, *p* <.0001), though this subgroup contained only one study, limiting its interpretability. For A2–B1 (*d* = − 0.3937, *p* =.1017) and B1–B2 levels (*d* = − 0.0399, *p* =.8630), human scores were slightly higher, but neither reached statistical significance.

When studies were grouped by the type of agreement index reported, some variation in effect sizes was observed. Studies reporting *Kappa* yielded a significant positive effect size (*d* = 0.2226, *p* =.0103), whereas studies reporting *Pearson’s r* (*d* = − 0.2329, *p* =.2253), *ICC* (*d* = 0.1846, *p* =.7388), *QWK* (*d* = − 0.5166, *p* =.2821), and *Spearman’s ρ* (*d* = − 0.3604, *p* =.7633) did not reach significance.

The number of raters analysis divided the studies into four groups: 1–2, 3–5, 6–15, and 16 + raters. No individual subgroup showed a statistically significant effect size (all *p* >.05). However, the meta-regression analysis revealed that AI-human scoring differences decreased as the number of raters increased (*β* = 0.0157, *p* =.0115). This apparent discrepancy reflects a methodological difference between the two analyses: meta-regression models the number of raters as a continuous predictor, capturing the overall linear trend, whereas subgroup analysis divides studies into discrete categories, reducing statistical power and obscuring the gradual effect.

The task type analysis compared speaking and writing tasks. No significant differences were observed for speaking (*d* = − 0.0180, *p* =.9319) or writing tasks (*d* = − 0.1613, *p* =.3967), with human ratings slightly higher than AI ratings in both cases.

## Discussion

### Magnitude of differences between AI and human scoring in language assessments (RQ1)

This study found no statistically significant difference between AI and human scoring, which carries practical implications for language assessments. When significant discrepancies arise between AI-generated and human-assigned scores, they may stem from either scoring source. On the human side, raters may be affected by cognitive biases, rubric interpretation drift, and order-related effects such as halo, proximity, and primacy effects^1,3,23,24^. On the AI side, systems trained on human-rated corpora may inherit biases from their training data and struggle with higher-order language features^26,27^. Significant discrepancies should therefore prompt investigation of both scoring sources rather than attribution to either one alone.

The pooled effect size suggests that the average systematic difference between AI and human scoring was small. Yet the near-total heterogeneity indicates that this average encompasses widely divergent outcomes. Purpose-built scoring systems (NLP/Hybrid, ML) showed minimal differences from human raters, while LLMs exhibited large discrepancies (*d*= −1.14). The overall non-significant result is therefore best understood as a central tendency across diverse conditions rather than a uniform finding. This result contrasts with optimistic claims in prior studies regarding small scoring differences (e.g^10,33^, and echoes Perelman’s^34^ argument that AI scoring systems should not be uncritically trusted or viewed as full substitutes for human judgment. While AI scoring is not yet capable of replicating nuanced human evaluation, it holds considerable potential as a “sentinel” for detecting human scoring anomalies and enhancing scoring reliability.

AI’s limitations necessitate assisted involvement of human raters to ensure the reliability of scores. This assumption aligns with the recommendations of Xi^15^ and Zou et al.^45^, which advocate for the collaboration between AI and human scoring, indicating that combining AI and human scoring provides a more comprehensive assessment of learners’ language abilities.

### Factors influencing the magnitude of differences between AI and human scoring (RQ2)

Despite advances in AI technology, the counterintuitive finding that AI-human scoring differences have increased over time invites careful consideration. Possible explanations are provided. First is the design orientation of AI systems. Earlier scoring engines such as E-rater and MY Access were developed explicitly for assessment purposes and trained on standardized tasks, thereby achieving small differences from human raters^41,43^. In contrast, recent LLMs like ChatGPT, while more linguistically sophisticated, are not optimized for scoring^14,35^. Second, LLMs are prompt-sensitive, and their output quality may vary depending on the user’s assessment literacy and prompt formulation skills^71,72^, introducing additional scoring variability not observed in earlier fixed-input systems. Therefore, the observed increase in scoring differences should not be interpreted as a technological regression, but rather as a consequence of the functional mismatch between general-purpose AI design and the specialized demands of automated assessment.

With respect to AI types, NLP + ML or DL + NLP+ML complex models were associated with significantly smaller differences between machine and human scoring. This indicates that more complex algorithms and model architectures are critical in reducing AI-human rating differences. Regarding the performance of LLMs, the subgroup analysis revealed that LLMs exhibited the largest differences from human scoring (*d* = −1.14, *k* = 5). This finding should be interpreted with caution given the small number of studies. While the lack of task-specific optimization is one plausible explanation^14,35^, other factors may also contribute, including variability in prompt design, mismatches between LLM output formats and scoring rubric scales, and differences in the training data underlying these models. Future research with a larger number of LLM-based studies is needed to disentangle these potential sources of discrepancy.

The finding of smaller AI-human scoring differences at CEFR B1–C1 levels compared to A2–B2 levels echoes previous findings that scoring differences decrease with higher learner proficiency^42^. This may be attributed to the greater structural stability and linguistic conformity of higher-level learner output, which allows AI systems to more easily identify features such as grammar, vocabulary, and coherence, thereby generating more stable scores^11^. In contrast, lower-level learners often produce language with frequent errors and semantic ambiguity, which reduces the accuracy of AI interpretation^24,46,73^. Learner proficiency therefore plays a critical role in shaping AI scoring effectiveness.

The observed reduction in AI-human scoring differences with a larger number of raters may stem from two key factors. First, AI systems, functioning as stable and multi-dimensional evaluators, provide concrete reference points that help raters calibrate their judgments, especially in ambiguous cases^15,32^. Second, more raters promote collaborative discussion and calibration around the scoring process^3,18^. This collaborative calibration helps standardize rubric interpretation and mitigate individual biases, ultimately reducing systematic differences between AI and human scores^25^.

The meta-regression and subgroup analyses indicated that the type of agreement index reported in primary studies was associated with variation in effect sizes. As these indices co-vary with other study-level design features, this pattern is best understood as reflecting methodological differences among studies. *Pearson’s r* measures the strength of linear association but does not account for systematic mean-level bias between scoring sources^10,47^. *Cohen’s Kappa*, while correcting for chance agreement, may behave unpredictably when score distributions are skewed or categories are unevenly distributed^20^. Xu et al.^47^, have argued that Limits of Agreement (*LOA*) may offer a more comprehensive assessment by capturing both systematic bias and individual-level variability. As each metric captures different aspects of scoring differences, researchers are encouraged to employ multiple complementary indices rather than relying on any single metric when evaluating AI-human scoring differences.

Although Egger’s test did not indicate statistically significant publication bias, slight funnel plot asymmetry and the sensitivity analysis results suggest that small-sample studies meaningfully influenced the overall estimate. Larger-sample studies tended to produce more stable results^26^, warranting cautious interpretation of the pooled effect size.

### Implications of AI-human scoring differences for language assessments (RQ3)

This study’s findings hold practical implications for the use of AI scoring systems in language assessment. The difference between AI and human scoring did not reach statistical significance, suggesting that AI scores may serve as a supplementary diagnostic reference for examining rating patterns.

Several moderating factors carry practical implications. Educators should prioritize AI scoring systems that have been specifically optimized for assessment tasks rather than simply adopting the latest general-purpose models. For lower-proficiency learners (CEFR A2–B1), whose language output tends to be less structured, human or hybrid scoring remains more appropriate, whereas AI scoring is more applicable at the B2–C1 levels due to greater linguistic stability. For complex or highly subjective tasks, such as creative writing or speaking interaction, human raters should remain involved, as AI may not fully capture deeper linguistic abilities. Increasing the number of raters can further reduce AI-human scoring differences by ensuring more uniform application of scoring criteria. When evaluating these differences, researchers are encouraged to report multiple complementary indices, combining both correlation-based and difference-based metrics, as no single index fully captures all dimensions of scoring differences^47^. Additionally, AI scoring systems validated on large samples tend to produce more stable results and should be preferred in standardized or large-scale assessment contexts.

Taken together, while AI scoring systems hold great potential in educational assessment, their effectiveness is context-dependent. Educators should consider these factors comprehensively when adopting AI scoring systems, strategically combining AI and human scoring to ensure fairness, accuracy, and efficiency in the scoring process.

## Conclusion

This study meta-analyzed 21 empirical studies to investigate differences between AI and human scoring in English writing and speaking assessments. The pooled difference did not reach statistical significance, though extremely high heterogeneity indicates that scoring differences are highly context-dependent. Purpose-built AI scoring systems produced scores close to human ratings, while general-purpose LLMs diverged considerably. When significant discrepancies arise, they may reflect issues in either or both scoring sources, positioning AI as a diagnostic reference tool. Several factors influenced the magnitude of scoring differences, including AI system type, publication year, CEFR level, number of raters, sample size, and the choice of agreement index.

Several limitations should be acknowledged. First, only 21 empirical studies were included, which may constrain the generalizability of the findings. Second, although this study investigated multiple moderating variables, it could not account for all potential factors influencing scoring differences, such as prompt design, scoring rubric characteristics, or rater training procedures. Third, Cohen’s d captures only systematic mean-level differences and does not assess the degree of agreement or reliability between AI and human scoring.

Three directions for future research are proposed. First, as AI scoring technologies continue to evolve rapidly, future meta-analyses should incorporate a larger and more representative body of literature. Primary studies should also adopt standardized reporting practices that include multiple complementary agreement indices, enabling more comprehensive quantitative synthesis across studies. Second, the effectiveness of AI scoring for lower-proficiency learners (CEFR A2–B1) warrants further investigation, as the relatively large scoring differences observed in this group suggest that AI systems struggle with unstructured and ambiguous language output. Third, hybrid scoring models that combine AI pre-scoring with human moderation should be empirically examined to determine whether such human-in-the-loop systems enhance scoring validity for complex tasks while maintaining efficiency in large-scale assessments.

## Data Availability

The data analyzed in this study were derived from previously published studies included in the meta-analysis. No new raw data were generated. The extracted datasets used for statistical analyses are not publicly available, as they were manually coded and compiled from multiple primary sources for the specific purpose of this study. However, the data are available from the corresponding author upon reasonable request.
